# Supplementary material for: Trends in Health Care Access/Experiences: Differential Gains across Sexuality and Sex Intersections before and after Marriage Equality
Source: Int J Environ Res Public Health. 2022 Apr 21;19(9):5075. doi: 10.3390/ijerph19095075 (PMC9101359; doi:10.3390/ijerph19095075)
Supplement: Supplementary file 1 [file ijerph-19-05075-s001.zip › ijerph-1659199-supplementary.pdf]

Supplement Table S1. Spearman's rank-sum correlations[1] between socioeconomic measures and time from years 2013 through 2018 (n=28,961).

	Correlation with Time
**Education Level[2]**	**0.12**
**Income[2]**	**0.20**
**Region**	
Northeast	0.03
Midwest	-0.00
South	-0.01
West	-0.01
**Employment**	
Full Time	-0.05
Part-Time	0.08
Retired	-0.07
Student/Homemaker	-0.02
Unemployed	0.03
**Marital Status**	
Single	**-0.12**
Married	**0.12**
Widowed	0.00
Divorced	-0.02
Separated	-0.01

[1]Point-biserial rank-sum correlations used for Region, Employment, and marital status.

[2]Education Level coded as Less than High School High School, College, Graduate Degree. Income coded as $24,999 or less, $25,000 to $49,000, $50,000 to $74,999, $75,000 to $99,999, $100,000 or more.

Correlations greater than or equal to 0.10 bolded.
